# *Lactiplantibacillus plantarum* A1, C1 and C10 Are Potential Probiotics Isolated from Pineapple Residual Silage

**DOI:** 10.3390/microorganisms11010029

**Published:** 2022-12-22

**Authors:** Hongbo Zeng, Yalu Liu, Kailang Huang, Hongwei Chen, Bin Yang, Jiakun Wang

**Affiliations:** 1 Institute of Dairy Science, College of Animal Sciences, Zhejiang University, Hangzhou 310058, China; 2 MoE Key Laboratory of Molecular Animal Nutrition, Zhejiang University, Hangzhou 310058, China

**Keywords:** lactic acid bacteria, probiotic function, antioxidant capacity, bovine jejunum epithelial cells, pineapple residual silage

## Abstract

The production and consumption of pineapple creates large quantities of residues. Ensiling these residues might help to minimize the waste burden and meet the intensive feed demand for ruminants. Proper lactic acid bacteria (LAB) are not only responsible for pineapple residual silage fermentation, but might also deliver probiotics. The aim of this study was to isolate LAB strains with probiotic functions, and to enhance intestinal antioxidant capacity from naturally fermented pineapple residues. A total of 47 LAB isolates with gram-positive, catalase-negative, nonhemolytic properties were used for probiotic screening. *Lactiplantibacillus plantarum* (*L. plantarum*) A1, C1 and C10 were susceptible to rifampicin, gentamicin and erythromycin, did not contain virulence factor-coding genes and showed good tolerance to acid (pH 3.0), 0.5% bile salt and simulated gastric and intestinal fluid. Their hydrophobicity indices were 71.92%, 45.50% and 66.90%, respectively. All of them were able to adhere to bovine jejunum epithelial cells (BJECs) and to antagonize *Escherichia coli* F5 and *Salmonella* Dublin. These three LAB strains tolerated hydrogen peroxide and significantly decreased (*p* < 0.05) reactive oxygen species levels in BJECs. In addition, *L. plantarum* C1 and C10 significantly increased (*p* < 0.05) the total antioxidant capacity in BJECs in the presence of 200 μmol/L hydrogen peroxide condition. *L. plantarum* A1, C1 and C10 are potential probiotics isolated from pineapple residual silage. This study aims to promote pineapple residue’s utilization in the feed industry.

## 1. Introduction

Pineapple (*Ananas comosus*) is a widely cultivated tropical fruit renowned not only for its unique aroma and sweetness, but also for its nutritional value and its anti-inflammatory and antioxidant activity [1]. More than 25 million tons of pineapple were produced worldwide in 2020 [2]. As it is composed of diverse chemical compounds, pineapple has been incorporated into various food products. The production and consumption of pineapple creates large quantities of residues. Byproducts from the pineapple industry can account for 70–75% *w*/*w* of the product, including the peel, core, crown, stem and leaf [3]. These residues are high in moisture, and biodegradable organic ingredients, if not disposed in time or unsuitably, will result in the waft of an unbearable stench during decomposition [4] and become breeding grounds for bacteria, pests and mice, thus leading to the spread of disease. On the other hand, these residues are also high in nutrients and are potential feed resources for ruminants. To help minimize the waste burden and meet the intensive feed demand from ruminants, ensiling these fruit residues for long-term storage is necessary. Lactic acid bacteria (LAB) converts sugar into lactic acid to inhibit the growth of the spoilage and pathogenic microorganisms [5], which could extend the preservation time of pineapple residuals. LAB fermentation can improve the content of riboflavin, folate, vitamin B12, sugar polymers, aroma compounds or low-calorie polyols (mannitol, sorbitol) in pineapple [6,7]. Thus, LAB are responsible for silage fermentation, and might also deliver probiotics and prebiotics to ruminants that consume pineapple residual silage.

The gastrointestinal microbiota of adult ruminants is rather stable and resilient, although probiotics and prebiotics are needed for early life. Calf diarrhea (also known as calf scouring) is a commonly reported disease and a major cause of economic loss for dairy farms [8]. Though more and more studies reported viral pathogens, such as BCoV, BoRVA was responsible for calf diarrhea [9,10], *Escherichia coli* F5 (K99) was the major diarrheal pathogenic bacteria of calves, while *Salmonella* fluctuated between different countries [11,12]. A high risk of calves being infected with pathogens has been associated with the negative effect of oxidative stress on immune responses [13]. More dramatic redox balance changes during calves’ first month of life were observed than in transition cows [14,15].

Therefore, we hypothesized that the LAB strains that were dominant in pineapple residual silages would have potential probiotic functions (especially the antibacterial activity against *Salmonella* spp. and *Escherichia coli*) and might enhance the intestinal antioxidant capacity. To test our hypothesis, in the present study, 96 LAB isolates were purified from naturally fermented pineapple residues. Their taxonomic status was studied through 16S rRNA gene sequencing. LAB with probiotic functions were gradually screened step by step according to the probiotic characteristics (peroxidase negative; nonhemolytic; no virulence gene; resistance to acid, bile salts and gastro-pancreatic digestion; inhibitory against *Escherichia coli* F5 (*E. coli* F5) and *Salmonella* Dublin (*S.* Dublin) in vitro; adhering to the surface of the bovine jejunum epithelial cells (BJECs); enhancing the antioxidant activity by BJECs model).

## 2. Materials and Methods

### 2.1. Silage Preparation

Fresh pineapple residues (peel, crown, and small part of pulp; Tainong 16, hybrid varieties, Taiwan, China) were collected from fruit stores on the Zhejiang campus, Zhejiang University (Hangzhou, Zhejiang, China). Residues were cut into 2 cm^2^ pieces and mixed evenly. Approximately 200 g of chopped residues in each of the three replicates was mixed without additives or supplements, vacuum-packed in a polyethylene bag (30 cm × 25 cm) and stored at room temperature (approximately 25 °C) for two months.

### 2.2. Isolation and Identification of LAB Strains

Pineapple residual silage samples (10 g) were blended with 90 mL sterilized phosphate-buffered saline (PBS) and shaken at 37 °C with 100 rpm agitation for 1 h (THZ-100B, Shanghai, China) to collect the supernatants. Immediately after collection, the supernatants were serially diluted 10^−3^ to 10^−5^ in sterilized PBS, plated on de Man, Rogosa and Sharpe (MRS) agar plates (Beijing Solaibao Technology Co. Ltd., Beijing, China) and incubated under anaerobic conditions (vinyl anaerobic chambers, Coy Laboratory Products, Grass Lake, MI, USA) at 37 °C for 24 h. Then, 10 to 20 strains were picked randomly from each plate, and a total of 96 isolates were collected. The gram stain appearance, catalase test and hemolytic activity of the isolates were determined as described by Kozaki et al. [16]. Their taxonomic status was classified using 16S rRNA gene sequencing. Total genomic DNA was extracted using the rapid bacterial genomic DNA isolation kit (Sangon Biotech, Shanghai, China) and amplified via PCR (polymerase chain reaction) using primers of 27F (5′-AGAGTTTGATCCTGGCTCAG-3′) and 1492R (5′- TACGGCTACCTTGTTACGACTT-3′). The PCR products were sequenced using the ABI 3730xl DNA analyzer (ABI Company, Tampa, FL, USA) at Sangon Biotech Co., Ltd. (Shanghai, China). The sequences obtained were compared using BLAST (basic local alignment search tool) and submitted to the GenBank sequence database to assign the strains [17]. A phylogenetic tree of the LAB strains was constructed using MEGA Version X software (version 11.0.13) [18], based on the adjacency method. 

### 2.3. Presence of Genes Encoding Virulence Factors

Adherent virulence factors *esp* (encoding enterococcal surface protein), *efaAfs* (cell wall adhesive), *asa* (aggregation substance) and *ace* (adhesion of collagen) aid the bacterium in adhesion and evasion of the host cell [19,20,21]; secretory virulence factors *gelE* (encoding gelatinase), *cylA* (cytolysin) and *hyl* (hyaluronidase) help the bacterium wade through the innate and adaptive immune response mounted within the host [22], and biogenic amine production factors *hdc* (encoding histidine decarboxylase), *tdc* (tyrosine decarboxylase) and *odc* (ornithine decarboxylase) act as biogenic amine production factors involved in biogenic amine production [23]. Therefore, the genes encoding virulence factors were detected using PCR with their specific primers (Table 1) and visualized using agarose gel (1%) electrophoresis, as described by Nagpal et al. [24]. In brief, predenaturation at 98 °C for 2 min, followed by 30 cycles (10 s of denaturation at 98 °C, 10 s of annealing at 57 °C, and 10 s of extension at 68 °C), with a final extension at 68 °C for 5 min. 

### 2.4. Anti-Pathogenic Activity

A lysogenic broth (LB) medium was used to grow *E. coli* F5 (BNCC125787) and *S.* Dublin (BNCC186358). The anti-pathogenic activity of the LAB isolates against *E. coli* F5 and *S.* Dublin was evaluated using the Oxford cup method [25]. Briefly, petri dishes (10 cm), containing 5 mL of LB agar and sterilized Oxford cups on top, were overlaid with 10 mL of soft LB agar seeded with 10^7^ CFU/mL pathogenic bacteria at 45 °C. After the medium solidified, the Oxford cup was removed, 150 μL of overnight culture LAB isolates was added to the wall and incubated at 37 °C for 16–18 h and the anti-pathogenic activity was determined by measuring the diameter of the inhibition zone around the LAB isolate spot.

### 2.5. Antibiotic Susceptibility

The antibiotic susceptibility of the LAB strains was assessed in a semiquantitative manner using the disc diffusion method of Charteris et al. [26], with 10^7^ CFU/mL of agar overlay. Antibiotic discs (Thermo Fisher Scientific, Shanghai, China) containing (per disc) ampicillin (10 µg), vancomycin (30 µg), gentamicin (10 µg), kanamycin (30 µg), streptomycin (10 µg), chloramphenicol (30 µg), erythromycin (15 µg), rifampicin (5 µg), amoxicillin (30 µg) and tetracycline (30 µg) were applied to agar plates inoculated with strains. After being cultivated at 37 °C for 24 h, the diameter of the inhibition zones was measured. Antibiotic susceptibility was recorded as susceptible (S), moderately susceptible (MS), or resistance (R), according to the interpretive criteria of *Enterobacteriaceae* in Performance Standards for Antimicrobial Disc Susceptibility Tests [27].

### 2.6. Cell Surface Hydrophobicity

The in vitro bacterial cell surface hydrophobicity of the LAB isolates was evaluated by measuring the microbial cell adhesion to hydrocarbons according to the method described by Nagpal et al. [24]. Briefly, the harvested cells (5000× *g* for 10 min) from the overnight culture were washed twice with PBS, and resuspended in PBS to 0.7 absorbance (Ab_s0_) at 600 nm. The mixture of 3 mL cell suspension and 0.6 mL n-hexadecane was vortexed for 2 min, and then incubated at 25 °C for 1 h to separate the aqueous and organic phases. The absorbance (Ab_s1_) of the aqueous phase was measured at 600 nm. The percentage of hydrophobicity was calculated by a decrease in absorbance, using the following formula: (1 − Ab_s1_/Ab_s0_) × 100.

### 2.7. Resistance to Gastrointestinal Conditions

#### 2.7.1. Tolerance to Low pH and Bile Salts

Adhesion of probiotics to the host epithelium is thought to increase competitive exclusion of pathogens [28]. There was a positive correlation between hydrophobicity and adhesion of probiotic bacteria [29]. Thus, only those strains with hydrophobicity higher than 40% were subjected to the assay of tolerance to low pH and bile salts. The tolerance of the LAB isolates to low pH bile salts, as well as continuous acid and bile, was assessed according to the method of Adetoye et al. [30], with minimal modifications. In brief, the cells were harvested (5000× *g* for 10 min) from the overnight culture and washed twice with PBS, then suspended and enumerated (N_0_, CFU/mL) in MRS. The resuspended cells were inoculated into fresh MRS broth; MRS broth adjusted to pH 4.0, 3.0 and 2.0; and fresh MRS broth containing 0%, 0.1%, 0.5% and 1% bile salts (Oxoid LP0055J, Thermo Fisher Scientific, Shanghai, China), respectively. After anaerobic incubation for 3 h at 37 °C, the cells were enumerated (N_t_, CFU/mL) again. Strain viability was calculated using the formula N_t_/N_0_ × 100. Continuous acid and bile tolerance was assessed at pH 3.0 for 3 h, followed by 1% bile salt for another 3 h of incubation. 

#### 2.7.2. Tolerance to Simulated Gastric and Intestinal Fluids

The tolerance to simulated gastrointestinal tract conditions was assessed following the method of Nagpal et al. [31], with the pepsin dissolved in sterile PBS, and the bile salts and pancreatin dissolved in PBS to simulate gastric fluid and intestinal fluid, respectively. The pH values used in the gastric and intestinal phases were adjusted to 3.0 and 8.0, respectively. The incubation times for the gastric and intestinal phases were both 6 h at 37 °C.

### 2.8. Inhibition of the Growth of E. coli F5 and S. Dublin

The *L. plantarum* A1, C1 and C10 were selected for pathogen co-culture experiments due to the above screenings. The rate of inhibition of the growth of *E. coli* F5 and *S.* Dublin by the three LAB strains was determined by a modified kinetic study of Adetoye et al. [30]. Briefly, a broth culture medium containing 5 mL of double strength MRS broth and 5 mL of double strength LB broth (MRS-LB) was prepared to support the growth of both LAB strains and *E. coli* F5 or *S.* Dublin. For the co-culture, the MRS-LB broth was inoculated with LAB and the test of *E. coli* F5 or *S.* Dublin both at 10^8^ CFU/mL was performed. Three experimental controls were set up, which consisted of LAB, *E. coli* F5 or *S.* Dublin as monocultures at 10^8^ CFU/mL. At 0, 8, 16 and 24 h, the cultures were counted on MRS agar, Salmonella–Shigella agar and eosin methylene blue agar.

### 2.9. Adhesion to BJECs

Similarly to the assays for bacterial adhesion to Caco-2 cells performed for probiotic screening in humans [32] and mice [33], the adhesion to BJECs, an immortalized BJEC cell line (Shanghai Saiqi Bioengineering Co. Ltd., Shanghai, China), was assessed in our screening. Briefly, cultured BJECs were grown in 12-well plates presented with round coverslips in Dulbecco’s modified Eagle medium (DMEM; Sangon, Shanghai, China) at 37 °C, with 5% CO_2_ in an incubator (Thermo, Waltham, MA, USA) until they reached over 80% confluence. Before the adhesion assay, the BJEC layers were washed twice with sterile PBS, suspended in 2 mL antibiotic-free and serum-free DMEM and incubated for 0.5 h. The overnight-grown LAB isolates were washed with PBS and suspended to 10^8^ CFU/mL with antibiotic-free and serum-free DMEM. Then, 100 µL of the LAB suspension was added to the BJEC cell wall, and incubated for another 2 h. After incubation, each wall was washed five times with PBS to remove nonadherent bacteria, and the round coverslips was picked out and fixed by drying in the incubator at 65 °C. After staining with safranin, the bacterial adhesion was observed under a microscope.

### 2.10. Antioxidant Capacity

#### 2.10.1. Survival Rate and Growth Curves at Different H_2_O_2_ Concentrations

The *L. plantarum* A1, C1 and C10 grown overnight were collected by centrifugation at 5000× *g* for 10 min, then washed twice with sterile PBS. They were then resuspended in fresh MRS broth containing 0, 2.0, 4.0, 6.0, 8.0 and 10.0 mmol/L H_2_O_2_, at a bacterial concentration of 10^8^ CFU/mL, and anaerobically incubated at 37 °C for 3 h. The survival rate was calculated by the viable *L. plantarum* cells before and after 3 h of incubation, and assessed by the plate count method.

For growth detection, the washed strains were resuspended in fresh MRS broth containing 0, 2.0, 4.0, 6.0, 8.0 or 10.0 mmol/L H_2_O_2_ to 0.4 initial absorbance (OD_600_). Then, 250 μL of the resuspended MRS broth was inoculated into 96-well microplates, and incubated at 37 °C. The OD_600_ of each well was measured (the microplate meter, SPARK, Tecan GmbH, Grödig, Austria) after vibrating (10 s) every 30 min for 48 h. For each strain, triplicates were performed for each concentration.

#### 2.10.2. Modulation of Reactive Oxygen Species (ROS) and Total Antioxidant Capacity (T-AOC) Activity of BJECs with or without H_2_O_2_

Similar to the adhesion assay, the *L. plantarum* A1, C1 and C10 were incubated with BJECs in 6-well microplates with 5% CO_2_ at 37 °C for 1 h. After that, the well H_2_O_2_ concentration was adjusted to 0 or 200 µmol/L for another 3 h of incubation. BJECs not inoculated with the *L. plantarum* A1, C1 and C10 were used as controls. Then, the total ROS levels, T-AOC activities and protein concentrations of the whole cell lysates (WCLs) in BJECs were measured using a CellRox^TM^ Deep Red Flow Cytometry Assay Kit (C10491; Thermo Fisher Scientific, Waltham, MA, USA), Total Antioxidant Capacity Assay Kit (A015-2; Nanjing Jiancheng Bioengineering Institute, Nanjing, China) and Pierce BCA Protein Assay Kit (Thermo Fisher Scientific, Shanghai, China), following the manufacturer’s protocol, respectively. The T-AOC activity (U/mL) was normalized to the total protein concentration (mg/mL) of the WCLs.

### 2.11. Statistical Analysis

For each isolate, triplicate data were acquired. Values are displayed as mean ± standard error. GraphPad Prism 8.0 software (La Jolla, CA, USA) was used to conduct one-way ANOVA for intracellular ROS content and T-AOC activity. The Tukey method was used for multiple comparisons between treatments. When *p* ≤ 0.05, the difference was significant; when 0.05 < *p* ≤ 0.1, it showed a trend of significance.

## 3. Results

### 3.1. Identification of the LAB Strains

**Figure 1:**
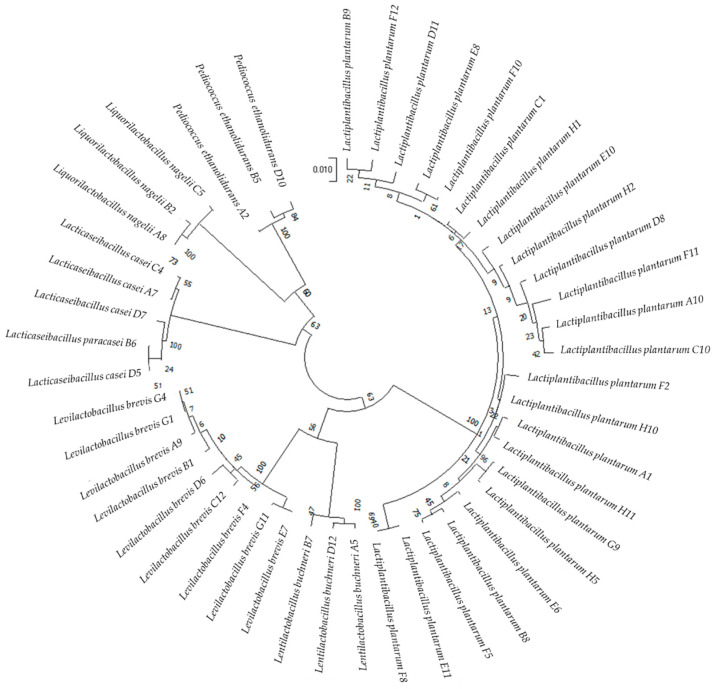
Circular phylogenetic tree showing the relative positions of the 47 LAB strains based on the neighbor-joining method of 16S rRNA genes with 1000 bootstrap replications.

Ninety five single colonies were gram-positive, catalase-negative and nonhemolytic LAB strains. Among these strains, 47 were unique, including 24 strains of *L. plantarum*; nine strains of *Levilactobacillus brevis*; four strains of *Lacticaseibacillus casei*; three each of *Liquorilactobacillus nagelii*, *Lentilactobacillus buchneri*, *Pediococcus ethanolidurans*; and one strain of *Lacticaseibacillus paracasei* (Figure 1).

### 3.2. Virulence Gene Detection

All 47 LAB strains were negative for *gelE*, *cylA*, *hyl*, *hdc*, *ace* and *tdc*, whereas 12 strains carried one, and seven strains carried two to three of these potential virulence genes (*esp*, *efaAfs*, *asa* or *odc*) (Table 2). 

### 3.3. Antibacterial Activity

The antibacterial activity of the 28 LAB strains without potential virulence genes was screened. *Levilactobacillus brevis* C12 and *Lentilactobacillus buchneri* D12 only inhibited the growth of *S.* Dublin, while 18 LAB strains inhibited (inhibition zone > 10 mm, Table 3) the growth of both *E. coli* F5 and *S.* Dublin. These included all the isolated 13 isolated strains of *L. plantarum*, one stain of *Pediococcus ethanolidurans*, two strains of *Liquorilactobacillus nagelii*, one stain of *Levilactobacillus brevis* and one strain of *Lentilactobacillus buchneri*.

### 3.4. Antibiotic Sensitivity

All 18 LAB strains with antibacterial activity towards both *E. coli* F5 and *S.* Dublin were sensitive to rifampicin, erythromycin, amoxicillin, ampicillin, tetracycline and chloramphenicol, and were resistant to vancomycin, streptomycin and kanamycin. *Levilactobacillus brevis* D6, *Lentilactobacillus buchneri* A5, *Liquorilactobacillus nagelii* A8 and *L. plantarum* F10 and H2 were resistant to gentamicin (Table 4). Therefore, 13 strains that were sensitive to gentamicin were screened for cell surface hydrophobicity. They were 11 strains of *L. plantarum*, and one each of *Liquorilactobacillus nagelii* and *Pediococcus ethanolidurans*.

### 3.5. Cell-Surface Hydrophobicity

The cell surface hydrophobicity of *Liquorilactobacillus nagelii* C5, *Pediococcus ethanolidurans* D10, and five strains of *L. plantarum* were lower than 10% (Table 5). The cell surface hydrophobicity of one *L. plantarum* strain (C1) was 45.50%, and that of the other four *L. plantarum* strains (A1, C10, E10, E11 and F8) ranged from 66.90% to 90.83%. 

### 3.6. Tolerance to Acids, Bile Salts and Gastric and Pancreatic Digestion

Six strains with cell surface hydrophobicity ≥ 45.50% were screened for gastrointestinal environment tolerance. As shown in Table 6 and Table 7, the survival rate of *L. plantarum* F8 decreased quickly from 259.38% (pH 7) to 53.02% (pH 4), to 13.40% (pH 3) and to 0.00% (pH 2). At pH 3, the other five *L. plantarum* strains (A1, C1, C10, E10 and E11) were able to retain a survival rate higher than 70%, but when the pH decreased to 2, *L. plantarum* E10 and E11 were completely inactivated. The 0.1% bile salt did not affect the survival rate of *L. plantarum* A1, C1 or C10, but exposure to 0.5% bile salt for 3 h decreased the survival rates of *L. plantarum* A1, C1 and C10 to 0.10%, 55.39% and 69.61%, respectively. Regarding exposure to 1% bile salt for 3 h, only 41.46% and 21.67% survival rates could be detected in *L. plantarum* C1 and C10, respectively. After incubation in simulated gastric conditions for 6 h, *L. plantarum* A1, C1 and C10 survived, with survival rates of 70.6%, 41.3% and 38.5%, respectively, but in simulated intestinal fluids, the survival of these strains was almost completely inhibited, whether for 3 or 6 h.

### 3.7. Inhibition of Growth of E. coli F5 and S. Dublin

**Figure 2:**
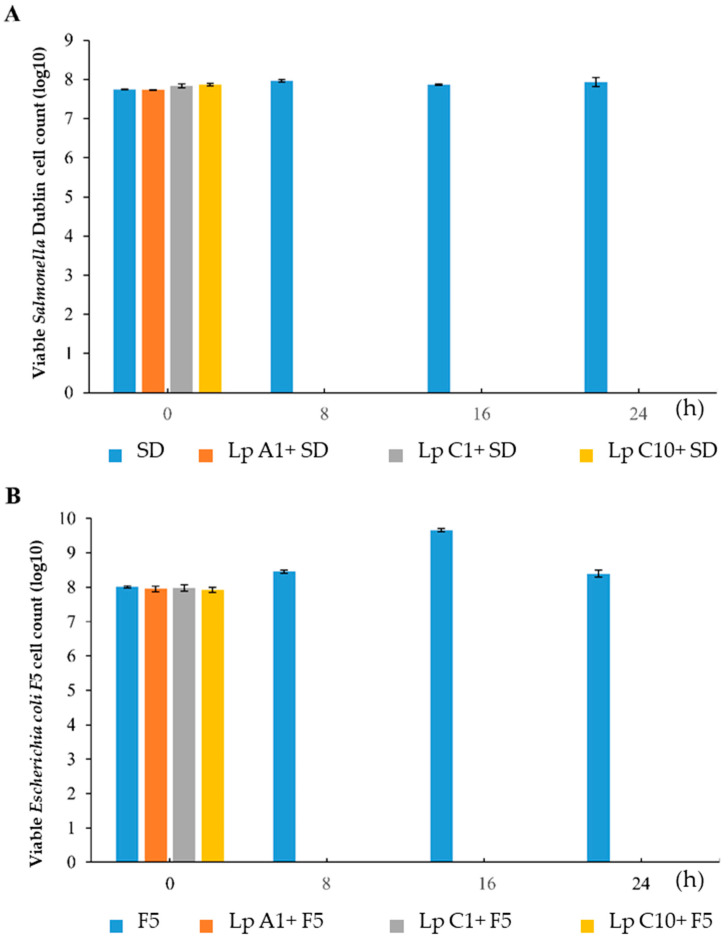
Monoculture or coculture of *Salmonella* Dublin (**A**) or *Escherichia coli* F5 (**B**) with *Lactiplantibacillus plantarum* A1, C1 and C10. SD = *Salmonella* Dublin; F5 = *Escherichia coli* F5; Lp = *Lactiplantibacillus plantarum*.

As shown in Figure 2, *E. coli* F5 and *S.* Dublin could not be counted in the co-cultures of *E. coli* F5 or *S.* Dublin with *L. plantrum* A1, C1 or C10 when sampled at 8, 16 and 24 h. 

### 3.8. In Vitro Adhesion to BJECs

**Figure 3:**
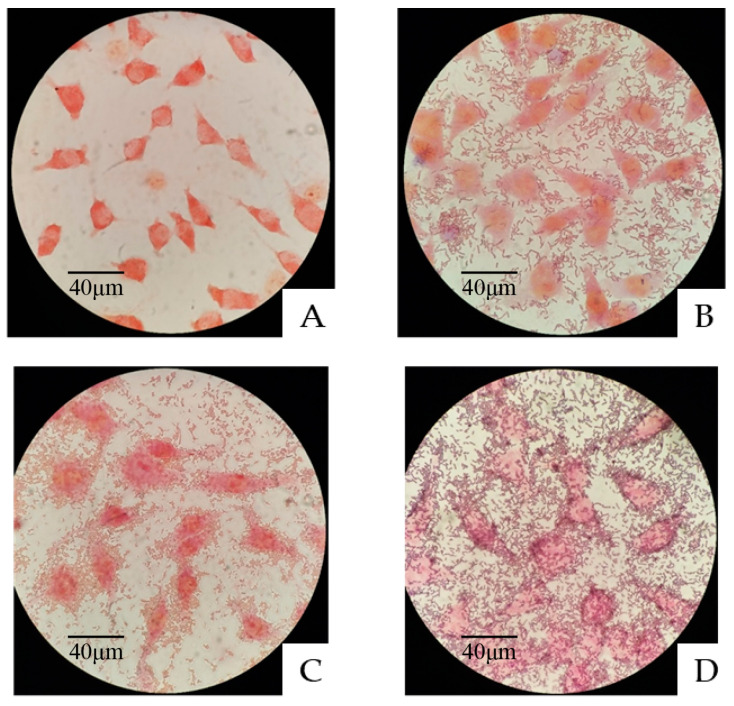
Adhesion of the three LAB strains to bovine jejunum epithelial cells (BJECs) observed under a light microscope. (**A**) Control BJECs. (**B**) Adhesion of *Lactiplantibacillus plantarum* A1 to the BJECs. (**C**) Adhesion of *Lactiplantibacillus plantarum* C1 to the BJECs. (**D**) Adhesion of *Lactiplantibacillus plantarum* C10 to the BJECs.

As shown in Figure 3, after 2 h of coincubation and five washes, significant amounts of *L. plantarum* A1, C1 or C10 remained around the BJECs, which indicated that the three strains of *L. plantarum* A1, C1 and C10 all had good cell adhesion.

### 3.9. Resistance of the Screened L. plantarum Strains to H_2_O_2_

**Figure 4:**
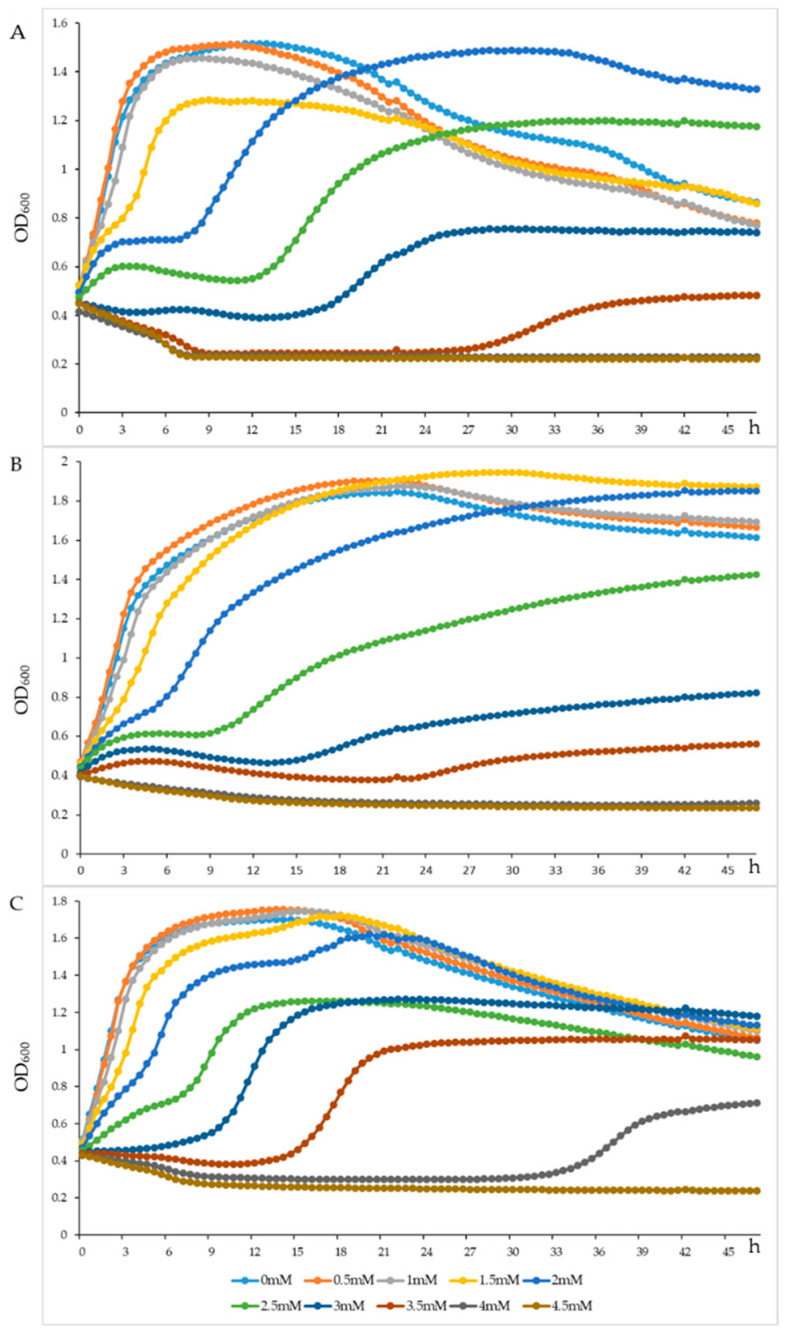
Growth density of the *Lactiplantibacillus plantarum* A1 (**A**), *Lactiplantibacillus plantarum* C1 (**B**) and *Lactiplantibacillus plantarum* C10 (**C**) strains under different concentration of H_2_O_2_. OD_600_ = the absorbance at 600 nm of the *Lactiplantibacillus plantarum* A1, C1 and C10.

The *L. plantarum* A1, C1 and C10 grew well, with a survival rate of more than 100% in MRS broth after incubation at 2 mmol/L H_2_O_2_ for 2 h (Table 8). However, their survival rate decreased to 0.5%, 0.06% and 12.7%, at 4 mmol/L H_2_O_2_, respectively, and was almost undetectable at 6, 8, and 10 mmol/L H_2_O_2_. The growth curves of *L. plantarum* A1 (Figure 4A), C1 (Figure 4B) and C10 (Figure 4C) incubated in MRS broth at 0 to 4.5 mmol/L H_2_O_2_, with 0.5 mmol/L as intervals, revealed that these three LAB strains could be resistant to 2 mmol/L H_2_O_2_ for more than 45 h.

### 3.10. Improving the Antioxidant Activity of BJECs

**Figure 5:**
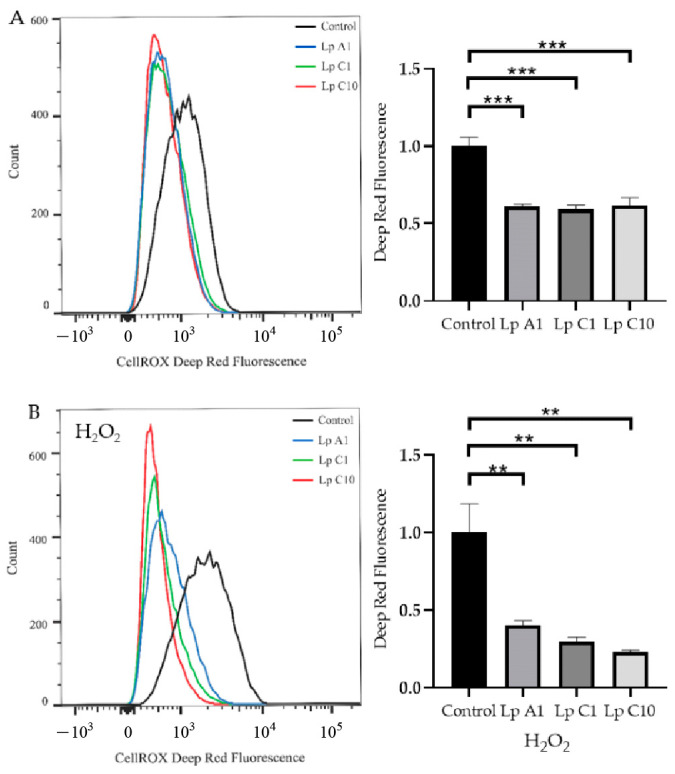
Reactive oxygen species (ROS) levels in bovine jejunum epithelial cells treated with *Lactiplantibacillus plantarum* A1, C1 and C10, under conditions without (**A**) or with (**B**) 200 μmol/L H_2_O_2_. Values are means ± standard error; ** *p* < 0.01, *** *p* < 0.001. Lp = *Lactiplantibacillus plantarum*.

**Figure 6:**
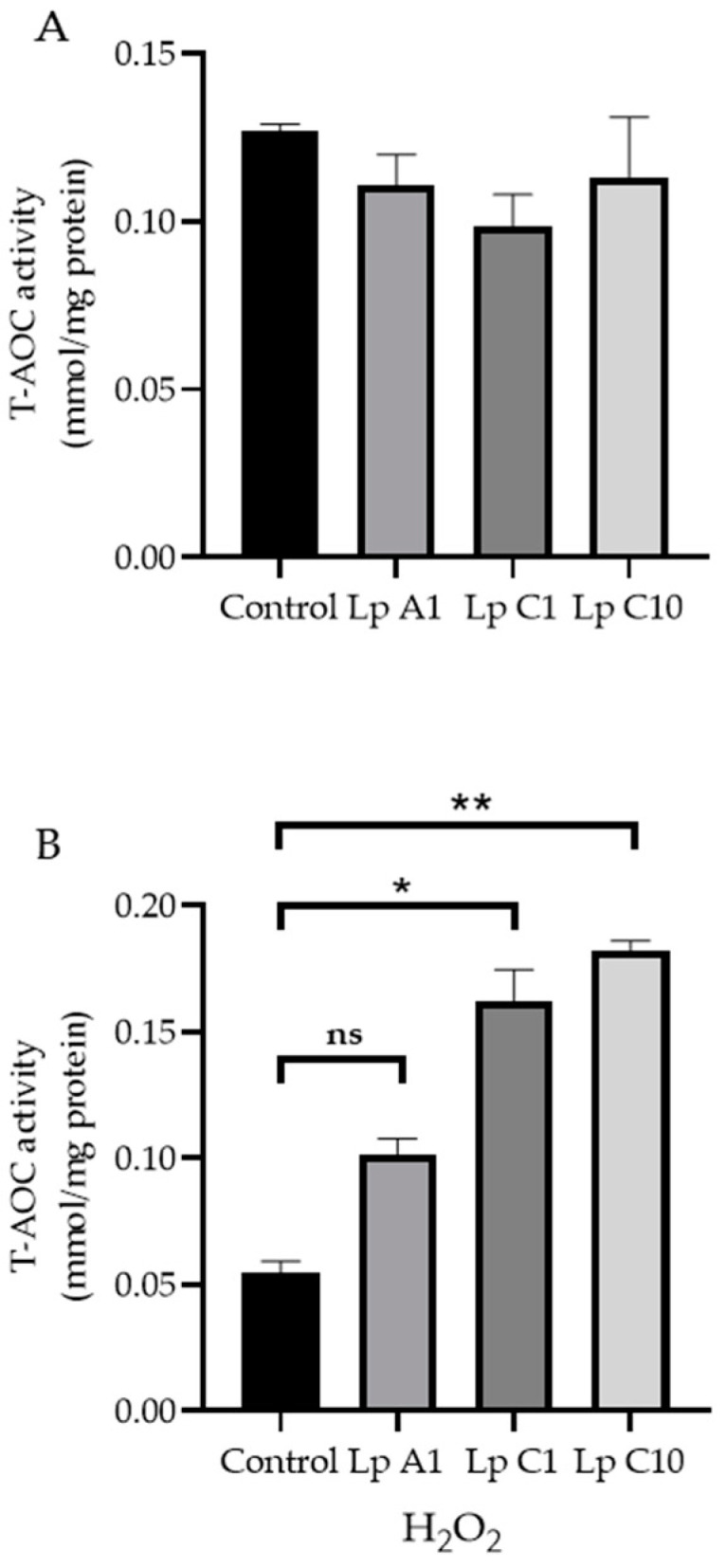
Total antioxidant capacity (T−AOC) activities in bovine jejunum epithelial cells treated with *Lactiplantibacillus plantarum* A1, C1 and C10, under conditions without (**A**) or with 200 μmol/L H_2_O_2_ (**B**). Values are means ± standard error; ns, not significant; * *p* < 0.05, ** *p* < 0.01. Lp = *Lactiplantibacillus plantarum*.

Compared to the control treatment, the coincubation of BJECs with *L. plantarum* A1, C1 or C10 significantly (*p* < 0.05) reduced ROS levels in BJECs with (Figure 5A) or without (Figure 5B) exposure to H_2_O_2_. However, the T-AOC activity was only increased in BJECs after exposure to H_2_O_2_ (Figure 6).

## 4. Discussion

Silage is an important part of ruminant diets. Ensiling pineapple residues provides not only carbohydrates, but also some functional fermentation products for feeding ruminants, while also reducing environmental pollution. The quality of silage is largely determined by LAB. For the purpose of delivering probiotics from ensiling pineapple residues, the LAB strains with antibiotic activity against *E. coli* F5 and *S* were used. Dublin (the major diarrheal pathogenic bacteria of calf diarrhea) was isolated from the naturally fermented pineapple residues to improve the antioxidant activity of intestinal tracts (highly associated with diarrheal pathogenic bacteria).

The LAB strains for utilization in feed products or other aspects should consider their safety, including detecting whether they contain virulence genes. *Esp, efaAfs, asa* and *ace* are adhesion virulence factors that can enhance the adhesion ability of pathogens to host cells, so as to infect the host and cause diseases [19,20,21]. *GelE, cylA* and *hyl* are secretory virulence factors. Pathogenic bacteria can degrade collagen and fibrin in the host body through the secretion, transfer and spread of gelatinase in the host body [22]. *Hdc, tdc* and *odc* can degrade histidine, tyrosine and ornithine into bioamines, and excessive bioamines in animal intestines will enter systemic circulation and pose a threat to host health [23]. Therefore, the isolated LAB should not include these virulence genes. 

Calf diarrhea is one of the major health challenges in cattle herds. The prevalence of *Salmonella* and *E. coli*, especially enter-toxigenic *E. coli* (ETEC), in diarrheic calves has been confirmed [34,35]. *S.* Dublin is a bovine-associated serotype of *Salmonella* that has strong adaptability in the calf intestinal tract. *E. coli* (BNCC125787) was isolated from the feces of diarrheal calves, encoding heat-stable enterotoxin and expressing F5 and F41 fimbriae. Both are the essential attributes of virulence for causing disease. Therefore, *E. coli* F5 (BNCC125787) and *S.* Dublin (BNCC186358) were used to screen the LAB strains with antimicrobial activity. In our study, of the 28 LAB strains, all the 13 *L. plantarum* strains had favorable antimicrobial activity against both *E. coli* F5 and *S.* Dublin. Similar results were also reported by Sharafi et al. [36]. In addition to *E. coli* and *Salmonella*, antimicrobial activity of the *L. plantarum* strains against *Pseudomonas* sp. and *Enterococcus faecalis* was reported [37]. Furthermore, in our study, *L. plantarum* A1, C1 and C10 inhibited the activity of *E. coli* F5 and *S.* Dublin during co-culture evaluation, which was consistent with the results of Abdel-Daim et al. [38].

Variable antibiotic sensitivities were recorded within strains of the same species. In our study, all the *L. plantarum* strains were susceptible to rifampicin, erythromycin, amoxicillin, ampicillin, tetracycline and chloramphenicol, but were resistant to streptomycin and vancomycin. These results were in agreement with Zhang and Karasu et al. [39,40], who found that the *L. plantarum* strains isolated from traditionally produced fermented vegetables showed resistance against vancomycin and streptomycin, and were consistent with the findings of Lee et al. [41], who studied the antibiotic susceptibility of *L. plantarum* isolated from kimchi, which showed susceptibility to ampicillin. Hummel et al. [42] also reported that the resistance to glycopeptides, such as vancomycin, is a common feature of LAB, especially *L. plantarum*. The vancomycin resistance of Lactobacilli has been reported to be intrinsic and chromosomally encoded [43,44]. However, the susceptibility to vancomycin is species-dependent and varies between species [45]. Hence, some resistant strains may harbor spontaneous mutations or acquired genes. Among the genes of the vancomycin resistance cluster, only the vanA gene is considered transferable via conjugation within the plasmid DNA [46] or the conjugative transposon [47]. With further study, we should amplify these vancomycin resistance clusters to explain whether the vancomycin resistance of our strains is acquired or intrinsic. Gentamicin is a strong bactericidal spectral antibiotic produced by the fermentation of *Rhodomonas* and *Echinospora,* and is widely used in clinical practice. *Levilactobacillus brevis* D6, *Lentilactobacillus buchneri* A5, *Liquorilactobacillus nagelii* A8, *L. plantarum* F10 and H2 were resistant to gentamicin, and these strains must be screened out from the candidates. 

The cell surface hydrophobicity helps in studying the colonization and adhesion of probiotic bacteria to epithelial cells in the gastrointestinal tract, which leads to the prevention of colonization by pathogens through their interaction [48]. A study that evaluated the probiotic properties and biological barrier resistance of 24 LAB strains showed that the percentage of hydrophobicity varied from 38.1% to 67.8% [49]. Del Re et al. [50] suggested that a minimum of 40.0% hydrophobicity is essential for a probiotic strain. Thus, the six *L. plantarum* strains with cell surface hydrophobicity levels higher than 45% were screened out in our study to ensure that these screened strains could colonize the epithelial cells and exert potentially probiotic functions in further application.

Probiotic microorganisms are required to be intrinsically tolerant to the stress factors prevalent in the gastrointestinal tract to ensure health benefits to the consumer [51]. Among the various stress factors, pH and bile salts are the most detrimental to the viability of probiotics. A pH of 3.0 is usually considered as an optimum pH for a successful probiotic to survive, due to the buffering impact of ingested food to raise pH to 3.0 in the stomach environment [52]. Five of the six selected *L. plantarum* strains with cell surface hydrophobicity survived well at pH 3.0, with a survival rate higher than 71%. In addition, bile salt has an antimicrobial impact on the intestinal microbiota [30]; therefore, it is essential for a probiotic to display tolerance to bile salt. For bile salts, a concentration between 0.3% and 0.5% is considered critical for the selection of potential probiotics [53]. *L. plantarum* strains (C1 and C10) survived well in 0.5% bile salts, with survival rates of 55.39% and 69.61%. The reason that *L. plantarum* could survive in 0.5%, and even 1% bile salts, might be because it contains bile salt hydrolase [54]. In addition, in our study, *L. plantarum* A1, C1 and C10 could also survive in simulated gastric and intestinal fluids for 6 h. As the *L. plantarum* A1, C1 and C10 strains demonstrated a strong ability to survive after exposure to the gastrointestinal tract conditions, they can be considered promising potential probiotic strains.

Adhesion of probiotics to the intestinal surface and colonization of the epithelial cells is an important requirement for probiotic activity. There is a positive correlation between hydrophobicity and adhesion ability [55]. *L. plantarum* A1, C1 and C10 passed the criterion of 45% hydrophobicity, and adhered closely to the cell surface after co-culture with the BJECs, which further indicated that these three strains had good cell adhesion and potential probiotic functions [56].

H_2_O_2_ participates in the formation of intracellular polar reactive oxygen molecules, and has good diffusion and persistence [57]. Compared with other free radicals, H_2_O_2_ is relatively mild and less toxic, but it can form more active ROSs, such as hydroxyl radicals, through the Fenton reaction and autoxidation reaction, thus damaging proteins, fats and DNA and causing oxidative damage to cells [57,58]. Increasing evidence has shown that probiotics, especially some LAB strains, exert important roles in reducing the accumulation of ROSs and improving antioxidant activities in the host [59,60]. In our study, we observed that *L. plantarum* A1, C1 and C10 were resistant to 2 mmol/L H_2_O_2_ for more than 45 h. Das et al. [61] observed that *L. plantarum* DM5 exhibited maximum resistance in the presence of 1.0 mmol/L H_2_O_2_, whereas *L. plantarum* AR113 could tolerate 8.0 mmol/L H_2_O_2_, and it entered the exponential phase at 48 h under 3.5 mmol/L H_2_O_2_. Thus, the three *L. plantarum* strains in our study showed favorable tolerance to H_2_O_2_ concentration.

Cell test results showed that the *L. plantarum* A1, C1 and C10 strains could reduce ROS accumulation in BJECs with or without H_2_O_2_ stimulation, and could increase T-AOC activity in BJECs with H_2_O_2_ stimulation. Therefore, all three of these strains had the ability to tolerate high H_2_O_2_ concentrations, which could reduce cellular oxidative stress by eliminating ROS in the extracellular environment of BJECs [62,63]. Infection of a calf’s intestinal tract with pathogenic bacteria has been associated with the negative effect of oxidative stress on immune responses [13]. In the present study, our results confirmed that the *L. plantarum* A1, C1 and C10 strains screened from the pineapple residues could reduce oxidative stress in a BJEC model.

## 5. Conclusions

*L. plantarum* A1, C1 and C10, isolated from pineapple residual silage, showed antibiotic activity against *E. coli* F5 and *S.* Dublin, the major pathogenic bacteria of diarrheal calves, and improved the antioxidant activity in BJECs. Therefore, *Lactiplantibacillus plantarum* A1, C1 and C10 are potential probiotics isolated from pineapple residual silage.

## Figures and Tables

**Table 1:** Primers used to detect the virulence factor-coding genes.

Virulence Genes and Products	Primers	Sequence (5’-3’)	Product (bp)	Tm °C
*gelE* (Gelatinase)	gelE-F	ACCCCGTATCATTGGTTT	419	56
	gelE-R	ACGCATTGCTTTTCCATC		
*cylA* (Cytolysin)	cylA-F	TGGATGATAGTGATAGGAAGT	517	57
	cylA-R	TCTACAGTAAATCTTTCGTCA		
*esp* (Enterococcal surface protein)	esp-F	TTGCTAATGCTAGTCCACGACC	933	62
	esp-R	GCGTCAACACTTGCATTGCCGAA		
*efaAfs* (Cell wall adhesins)	efaAfs-F	GACAGACCCTCACGAATA	705	52
	efaAfs-R	AGTTCATCATGCTGTAGTA		
*hyl* (Hyaluronidase)	HYLn1	ACAGAAGAGCTGCAGGAAATG	276	56
	HYLn2	GACTGACGTCCAAGTTTCCAA		
*asa* (Aggregation substance)	ASA11	GCACGCTATTACGAACTATGA	375	56
	ASA12	TAAGAAAGAACATCACCACGA		
*hdc* (Histidine decarboxylase)	JV16HC	AGATGGTATTGTTTCTTATG	367	52
	JV17HC	AGACCATACACCATAACCTT		
*ace* (Adhesion of collagen)	ACE-F	GAATTGAGCAAAAGTTCAATCG	1008	55
	ACE-R	GTCTGTCTTTTCACTTGTTTC		
*tdc* (Tyrosine decarboxylase)	P2-for	GAYATNATNGGNATNGGNYTNGAYCARG	924	52
	P1-rev	CCRTARTCNGGNATAGCRAARTCNGTRTG		
*odc* (Ornithine decarboxylase)	odc-3	GTNTTYAAYGCNGAYAARACNTAYTTYGT	1446	52
	odc-16	ATNGARTTNAGTTCRCAYTTYTCNGG		

**Table 2:** Virulence gene detection in the LAB strains.

Strains	Virulence Genes									
	* gelE *	* cylA *	* esp *	* efaAfs *	* hyl *	* asa *	* hdc *	* ace *	* tdc *	* odc *
*Lactiplantibacillus plantarum* A1	−	−	−	−	−	−	−	−	−	−
*Lactiplantibacillus plantarum* A10	−	−	+	+	−	−	−	−	−	−
*Lactiplantibacillus plantarum* B8	−	−	+	+	−	+	−	−	−	−
*Lactiplantibacillus plantarum* B9	−	−	+	−	−	−	−	−	−	−
*Lactiplantibacillus plantarum* C1	−	−	−	−	−	−	−	−	−	−
*Lactiplantibacillus plantarum* C10	−	−	−	−	−	−	−	−	−	−
*Lactiplantibacillus plantarum* D11	−	−	+	−	−	+	−	−	−	−
*Lactiplantibacillus plantarum* D8	−	−	+	−	−	−	−	−	−	−
*Lactiplantibacillus plantarum* E10	−	−	−	−	−	−	−	−	−	−
*Lactiplantibacillus plantarum* E11	−	−	−	−	−	−	−	−	−	−
*Lactiplantibacillus plantarum* E6	−	−	−	+	−	+	−	−	−	−
*Lactiplantibacillus plantarum* E8	−	−	+	−	−	−	−	−	−	−
*Lactiplantibacillus plantarum* F10	−	−	−	−	−	−	−	−	−	−
*Lactiplantibacillus plantarum* F11	−	−	−	−	−	−	−	−	−	−
*Lactiplantibacillus plantarum* F12	−	−	−	−	−	−	−	−	−	−
*Lactiplantibacillus plantarum* F2	−	−	+	−	−	−	−	−	−	−
*Lactiplantibacillus plantarum* F5	−	−	+	+	−	+	−	−	−	−
*Lactiplantibacillus plantarum* F8	−	−	−	−	−	−	−	−	−	−
*Lactiplantibacillus plantarum* G9	−	−	−	−	−	−	−	−	−	−
*Lactiplantibacillus plantarum* H1	−	−	+	−	−	−	−	−	−	−
*Lactiplantibacillus plantarum* H10	−	−	−	−	−	−	−	−	−	−
*Lactiplantibacillus plantarum* H11	−	−	+	−	−	−	−	−	−	−
*Lactiplantibacillus plantarum* H2	−	−	−	−	−	−	−	−	−	−
*Lactiplantibacillus plantarum* H5	−	−	−	−	−	−	−	−	−	−
*Levilactobacillus brevis* A9	−	−	+	+	−	−	−	−	−	−
*Levilactobacillus brevis* B1	−	−	−	−	−	−	−	−	−	−
*Levilactobacillus brevis* C12	−	−	−	−	−	−	−	−	−	−
*Levilactobacillus brevis* D6	−	−	−	−	−	−	−	−	−	−
*Levilactobacillus brevis* E7	−	−	−	−	−	−	−	−	−	−
*Levilactobacillus brevis* F4	−	−	−	−	−	−	−	−	−	−
*Levilactobacillus brevis* G1	−	−	−	−	−	−	−	−	−	−
*Levilactobacillus brevis* G11	−	−	−	−	−	−	−	−	−	−
*Levilactobacillus brevis* G4	−	−	−	−	−	−	−	−	−	−
*Lacticaseibacillus casei* A7	−	−	−	+	−	−	−	−	−	−
*Lacticaseibacillus casei* C4	−	−	−	+	−	−	−	−	−	−
*Lacticaseibacillus casei* D5	−	−	−	+	−	−	−	−	−	−
*Lacticaseibacillus casei* D7	−	−	−	+	−	−	−	−	−	−
*Liquorilactobacillus nagelii* A8	−	−	−	−	−	−	−	−	−	−−
*Liquorilactobacillus nagelii* B2	−	−	−	−	−	−	−	−	−	−
*Liquorilactobacillus nagelii* C5	−	−	−	−	−	−	−	−	−	−
*Lentilactobacillus buchneri* A5	−	−	−	−	−	−	−	−	−	−
*Lentilactobacillus buchneri* B7	−	−	−	−	−	−	−	−	−	−
*Lentilactobacillus buchneri* D12	−	−	−	−	−	−	−	−	−	−
*Lacticaseibacillus paracasei* B6	−	−	−	−	−	−	−	−	−	+
*Pediococcus ethanolidurans* A2	−	−	+	+	−	−	−	−	−	−
*Pediococcus ethanolidurans* B5	−	−	+	−	−	−	−	−	−	−
*Pediococcus ethanolidurans* D10	−	−	−	−	−	−	−	−	−	−

+/− means contained or nor contained corresponding virulence factor-coding genes.

**Table 3:** Antagonistic activity of the LAB strains against *Escherichia coli* F5 and *Salmonella* Dublin.

Strains	Diameter of Inhibition (mm)	
	*E. coli* F5	*S.* Dublin
*Levilactobacillus brevis* B1	−	−
*Levilactobacillus brevis* C12	−	+
*Levilactobacillus brevis* D6	+	+
*Levilactobacillus brevis* E7	−	−
*Levilactobacillus brevis* F4	−	−
*Levilactobacillus brevis* G1	−	−
*Levilactobacillus brevis* G11	−	−
*Levilactobacillus brevis* G4	−	−
*Lentilactobacillus buchneri* A5	+	+
*Lentilactobacillus buchneri* B7	−	−
*Lentilactobacillus buchneri* D12	−	+
*Liquorilactobacillus nagelii* A8	+	+
*Liquorilactobacillus nagelii* B2	−	−
*Liquorilactobacillus nagelii* C5	+	+
*Lactiplantibacillus plantarum* A1	+	+
*Lactiplantibacillus plantarum* C1	+	+
*Lactiplantibacillus plantarum* C10	+	+
*Lactiplantibacillus plantarum* E10	+	+
*Lactiplantibacillus plantarum* E11	+	+
*Lactiplantibacillus plantarum* F10	+	+
*Lactiplantibacillus plantarum* F11	+	+
*Lactiplantibacillus plantarum* F12	+	+
*Lactiplantibacillus plantarum* F8	+	+
*Lactiplantibacillus plantarum* G9	+	+
*Lactiplantibacillus plantarum* H10	+	+
*Lactiplantibacillus plantarum* H2	+	+
*Lactiplantibacillus plantarum* H5	+	+
*Pediococcus ethanolidurans* D10	+	+

+/− means diameter of inhibition above or less than 10 mm.

**Table 4:** Antibiotic susceptibility of the LAB strains.

Strains	Antibiotic ^1^									
	RIF	VAN	GEN	STR	KAN	ERY	AMO	AMP	TET	CHL
*Levilactobacillus brevis* D6	S	R	R	R	R	S	S	S	S	S
*Lentilactobacillus buchneri* A5	S	R	R	R	R	S	S	S	S	S
*Liquorilactobacillus nagalii* C5	S	R	S	R	R	S	S	S	S	S
*Liquorilactobacillus nagelii* A8	S	R	R	R	R	S	S	S	S	S
*Lactiplantibacillus plantarum* A1	S	R	S	R	R	S	S	S	MS	S
*Lactiplantibacillus plantarum* C1	S	R	S	R	R	S	S	S	MS	S
*Lactiplantibacillus plantarum* C10	S	R	S	R	R	S	S	S	MS	S
*Lactiplantibacillus plantarum* E10	S	R	S	R	R	S	S	S	MS	S
*Lactiplantibacillus plantarum* E11	S	R	S	R	R	S	S	S	S	S
*Lactiplantibacillus plantarum* F10	S	R	R	R	R	S	S	S	S	S
*Lactiplantibacillus plantarum* F11	S	R	S	R	R	S	S	S	S	S
*Lactiplantibacillus plantarum* F12	S	R	S	R	R	S	S	S	S	S
*Lactiplantibacillus plantarum* F8	S	R	S	R	R	S	S	S	MS	S
*Lactiplantibacillus plantarum* G9	S	R	S	R	R	S	S	S	MS	S
*Lactiplantibacillus plantarum* H10	S	R	S	R	R	S	S	S	MS	S
*Lactiplantibacillus plantarum* H2	S	R	R	R	R	S	S	S	S	S
*Lactiplantibacillus plantarum* H5	S	R	S	R	MS	S	S	S	S	S
*Pediococcus ethanolidurans* D10	S	R	S	MS	R	S	S	S	MS	S

^1^ RIF = rifampicin; VAN = vancomycin; GEN = gentamicin; STR = streptomycin; KAN = kanamycin; ERY = erythromycin; AMO = amoxicillin; AMP = ampicillin; TET = tetracycline; CHL = chloramphenicol. S = susceptible; R = resistant; MS = moderately susceptible.

**Table 5:** Cell surface hydrophobicity of the LAB strains.

Strains	Cell Surface Hydrophobicity (%)
*Liquorilactobacillus nagelii* C5	9.61 ± 0.79 ^d^
*Lactiplantibacillus plantarum* A1	71.92 ± 1.51 ^b^
*Lactiplantibacillus plantarum* C1	45.50 ± 1.45 ^c^
*Lactiplantibacillus plantarum* C10	66.90 ± 2.93 ^b^
*Lactiplantibacillus plantarum* E10	90.83 ± 1.68 ^a^
*Lactiplantibacillus plantarum* E11	86.24 ± 0.51 ^a^
*Lactiplantibacillus plantarum* F11	6.37 ± 2.23 ^d^
*Lactiplantibacillus plantarum* F12	4.66 ± 0.74 ^d^
*Lactiplantibacillus plantarum* F8	85.99 ± 2.28 ^a^
*Lactiplantibacillus plantarum* G9	3.92 ± 0.47 ^de^
*Lactiplantibacillus plantarum* H10	1.92 ± 0.28 ^e^
*Lactiplantibacillus plantarum* H5	7.45 ± 1.36 ^d^
*Pediococcus ethanolidurans* D10	9.27 ± 1.39 ^d^

^a–e^ Different superscripts within a column indicate a significant difference at the 0.05 level.

**Table 6:** Survivability of the LAB strains in acidic pH and bile salts.

Strains	Survival Rate (%)						
	pH = 7	pH = 4	pH = 3	pH = 2	Bile Salt = 0.1%	Bile Salt = 0.5%	Bile Salt = 1%
*Lactiplantibacillus plantarum* A1	766.45 ± 53.32 ^b^	194.38 ± 10.88 ^b^	79.34 ± 5.14 ^b^	10.27 ± 2.590 ^a^	106.59 ± 7.090 ^b^	0.10 ± 0.01 ^d^	0.03 ± 0.01 ^c^
*Lactiplantibacillus plantarum* C1	324.75 ± 6.810 ^d^	172.30 ± 7.700 ^c^	89.46 ± 4.58 ^b^	5.17 ± 0.87 ^b^	115.44 ± 11.14 ^b^	55.39 ± 1.400 ^b^	41.46 ± 2.800 ^a^
*Lactiplantibacillus plantarum* C10	350.00 ± 13.34 ^d^	235.78 ± 9.660 ^a^	135.29 ± 13.66 ^a^	0.50 ± 0.10 ^c^	111.76 ± 5.160 ^b^	69.61 ± 7.400 ^a^	21.67 ± 1.480 ^b^
*Lactiplantibacillus plantarum* E10	637.55 ± 29.21 ^c^	186.62 ± 12.18 ^b^	83.46 ± 2.28 ^b^	0.00 ^c^	135.13 ± 14.36 ^a^	52.38 ± 2.720 ^b^	1.88 ± 0.16 ^c^
*Lactiplantibacillus plantarum* E11	1039.58 ± 102.15 ^a^	210.42 ± 13.03 ^a^	71.46 ± 3.00 ^b^	0.00 ^c^	117.11 ± 0.820 ^b^	1.95 ± 0.10 ^d^	0.12 ± 0.00 ^c^
*Lactiplantibacillus plantarum* F8	259.38 ± 14.27 ^e^	53.02 ± 2.20 ^d^	13.40 ± 0.98 ^c^	0.00 ^c^	34.45 ± 3.05 ^c^	10.51 ± 0.340 ^c^	0.65 ± 0.12 ^c^

^a–e^ Different superscripts within each column indicate a significant difference at the 0.05 level.

**Table 7:** Viability of the LAB strains after exposure to simulated gastric and intestinal fluids.

Strains	Survival Rate (%)		
	Gastric Fluid	Intestinal Fluid for 3 h	Intestinal Fluid for 6 h
*Lactiplantibacillus plantarum* A1	70.6 ± 6.63 ^a^	0.05 ± 0.01 ^b^	0.03 ± 0.01 ^b^
*Lactiplantibacillus plantarum* C1	41.3 ± 1.77 ^b^	0.8 ± 0.04 ^b^	0.7 ± 0.02 ^b^
*Lactiplantibacillus plantarum* C10	38.5 ± 1.48 ^b^	4.1 ± 0.41 ^a^	3.7 ± 0.12 ^a^

^a,b^ Different superscripts within each column indicate a significant difference at the 0.05 level.

**Table 8:** Survival rates (%) of *Lactiplantibacillus plantarum* A1, C1 and C10 in different concentrations of H_2_O_2_.

Concentration of H_2_O_2_ (mmol/L)	Survival Rate (%)		
	*L. plantarum* A1	*L. plantarum* C1	*L. plantarum* C10
0	345.0 ± 31.9 ^b^	342.6 ± 28.85 ^b^	366.7 ± 26.67 ^a^
2	172.7 ± 15.36 ^a^	146.4 ± 12.95 ^b^	182.7 ± 7.69 ^a^
4	0.5 ± 0.18 ^b^	0.06 ± 0.017 ^b^	12.7 ± 6.61 ^a^
6	<0.01 ^a^	<0.01 ^a^	<0.01 ^a^
8	<0.01 ^a^	<0.01 ^a^	<0.01 ^a^
10	0 ^a^	0 ^a^	<0.01 ^a^

^a,b^ Different superscripts within each row indicate a significant difference at the 0.05 level.

## Data Availability

The sequenced data reported in the current study have been deposited in the GenBank (Accession No. OQ096519-OQ096614).
